# Antidepressant Effect and Mechanism of *Picea mariana* Essential Oil on Reserpine-Induced Depression Model Mice

**DOI:** 10.4014/jmb.2405.05013

**Published:** 2024-07-18

**Authors:** Ying Wang, Guofeng Shi, Yixi Zeng, Juting Li, Yongyu Wu, Jiahui Zheng, Anjing Xu, Yanqing Ma, Lanyue Zhang, Hui Li

**Affiliations:** 1 School of Biomedical and Pharmaceutical Sciences, Guangdong University of Technology, Guangdong Provincial Key Laboratory of Plant Resources Biorefinery, Guangzhou 510006, P.R. China; 2 Department of Traditional Chinese Medicine, Institute of Guangdong Geriatric, Guangdong Provincial People's Hospital (Guangdong Academy of Medical Sciences), Southern Medical University Guangzhou 510080, P.R. China

**Keywords:** *Picea mariana*, essential oil, reserpine-induced depression, neurotransmitter, nervous system

## Abstract

The disturbance of brain biochemical substances serves as a primary cause and aggravating factor of depression. This study aimed to investigate the principal components of *Picea mariana* and its effect on reserpine-induced depression mice,w ith its relationship with brain central transmitters and related proteins. The main constituents of *P. mariana* essential oil (PMEO) were analyzed by GC-MS spectrometry. The quiescent time in the tail suspension test (TST) and forced swim test (FST), along with the weight change of the mice was detected. The number of normal neurons was quantified through Nissl staining. Immunohistochemistry was employed to determine the levels of 5HT-_1A_ and 5HT-_2A_ in the brain. Western blotting was utilized to detect 5HT-_2A_, CRF and TrkB protein levels. RT-qPCR was used to detect the mRNA levels of 5HT-_1A_, 5HT-_2A_, TrkB, CRF, and BDNF. The main active ingredients of PMEOs were (-) -bornyl acetate (44.95%), γ-Terpinene (14.17%), and β-Pinene (10.12%). PMEOs effectively improved the retardation and weight loss due to anorexia in depression-like mice. This improvement was associated with an increase in the number of normal neurons. After administering different doses of PMEOs, the levels of 5HT-_1A_, 5HT-_2A_, CRF, and TrkB were found to be increased in brain tissue. RT-qPCR revealed that the mRNA levels of CRF, 5HT-_1A_, and 5HT-_2A_ were generally upregulated, whereas TrkB and BDNF were downregulated. PMEO can effectively alleviate depression induced by reserpine, which may be attributed to its regulation of 5HT-_1A_, 5HT-_2A_, CRF and TrkB protein expression, thus reducing brain nerve injury.

## Introduction

Major depressive disorder (MDD) is a prevalent psychological disorder that is expected to overtake most of the other causes of disability globally. The presents are negative mood, diminished interest or pleasure, low energy or self-worth, trouble sleeping or eating, and difficulty concentrating [1]. Today, 350 million individuals are thought to be affected by depression (WHO, 2012). Nevertheless, the mechanism that explains the incidence of depression and the effects of antidepressants remains unclear. Research about depression has mainly examined how monoamine neurotransmitters interact with receptor proteins and their reuptake [2]. The majority of current chemosynthetic antidepressants are ineffective for many patients and have a high risk of complicated adverse effects [3]. Researching and developing antidepressants that are effective while having fewer side effects is therefore extremely important. It is essential to take into consideration additional mechanisms of depression development as they may offer prospective therapeutic targets for depression that are more successful and may have fewer side effects [4].

*Picea mariana* (Mill.) Britton, Sterns & Poggenb. and its derivatives have been widely used in the biomedical field with various health benefits. *P. mariana* can isolate various bioactive compounds in the steam distillation process, which has better effects than other products for some diseases or conditions. Numerous biological properties of *P. mariana* have been documented, including immunomodulatory, anti-inflammatory, and antioxidant activities [5, 6]. On the other hand, absolutely nothing has been discovered about how *P. mariana* essential oils (PMEOs) affect neuroinflammatory indicators and immune-triggered behavioral abnormalities. Interestingly, while anti-inflammatory or antidepressant effects of PMEOs have recently been demonstrated in mice, these observations were made in chronic stress models, and there is not any clear pharmacological proof linking PMEOs to immune-triggered depression [7]. In this study, a reserpine-induced depressive-like mice model was used to examine the potential mechanism and effect of PMEOs on depressive behavior caused by neurotransmitter disorders.

5HT in the brain has been linked to the pathophysiology and treatment of psychiatric disorders, and serotonin acts by interacting with a protein-coupled receptor family of multiple membrane-binding receptor families [8]. The 5HT-_1A_ and 5HT-_2A_ receptors are the most widely distributed 5HT receptor subtype expressed in neuronal regions, including the hippocampus, cerebral cortex, and hypothalamus, and are involved in the pathogenesis of anxiety, depression, and mood regulation [9, 10]. It has been found that downregulation of 5HT-_1A_ and 5HT-_2A_ receptor expression in postsynaptic increases depressive behavior [11], while activation of these receptors by antidepressants could decrease it. Depression is mainly associated with loss of 5HT-_2A_ in the synaptic cleft and abnormal activity of corticotrophin-releasing factor (CRF) [12, 13]. Brain-derived neurotrophic factor (BDNF), is a crucial neurotrophic factor affecting the survival, differentiation, and growth of normal neurons. Tyrosine kinase receptor B (TrkB) is a receptor for BDNF, and TrkB signaling may be activated under conventional antidepressant therapy [14]. In this study, immunofluorescence, Western blotting and RT-qPCR were used to investigate the regulatory effects of these neurotransmitter indicators after PMEOs treatment.

## Materials and Methods

### Essential Oils and Chemicals

The dried bark of *P. mariana* (Mill.) Britton, Sterns & Poggenb. was purchased from the Traditional Chinese Medicine Tong Ren Tang Group Co., Ltd. and was identified by Professor Nian Liu (Zhongkai University of Agriculture and Engineering). *P. mariana* essential oil was extracted from the dried bark by steam distillation. The specimen of the PMEOs (No. 2021-101P) was stored in the Institute of Nature Medicine and Green Chemistry (Guangdong University of Technology).

First, the dried bark was pulverized into powder with a 0.45 mm diameter. The powder was then put in a Clevenger-type device, and the extraction time was 4 h. Above the water layer, the essential oil was gathered and dried with a small amount of anhydrous sodium sulfate. At last, the essential oil was kept at 4°C for later research. The following formula was used to determine the essential oil yield:

Essential oil yield (%) = Mass of essential oil (g)/ Mass of extracted plant material powder (g)

### Gas Chromatography-Mass Spectrometry Analyses (GC-MS)

The chemical composition of *P. mariana* was analyzed under the following conditions (Thermo Electron Corp., USA): The chromatographic column was DB-WAX capillary column (60m×0.25mm×0.25μm). Heating procedure: The initial temperature was kept at 70°C for 2 min, then gradually raised to 120°C at a rate of 3°C/min, then to 230°C at a rate of 4°C/min, and maintained at the temperature for 5 min. The linear velocity of the helium carrier gas was 1.0 ml/min, and the temperature of the gasification chamber was 260°C. Besides the electron bombardment ion source, the electron energy was 70 eV; The transmission line temperature was 280°C; The ion source temperature was 230°C; The quadrupole temperature was 150°C. Besides, the retention indices (RIs) of each compound were calculated with a homologous series of n-alkanes (C_6_-C_40_) standards (Fattahi *et al*., 2016). The component identification of essential oils was performed by comparing mass spectra to published data with NIST Chemistry libraries.

### Animal Models

Fifty-four 5-week-old male KM mice weighing 20 (±2) g were acquired from the Guangdong Experimental Animal Center, which was performed on a 12-h light/dark cycling in a temperature-controlled (25 ± 1°C) colony and had free access to water and food ad libitum. Before the beginning of the experiment, mice were allowed to adapt to laboratory conditions for one week. The experimental protocols for animal ethics were approved by the Laboratory Animal Centre of Sun Yat-Sen University (Approval Document: SCXK/20130002). To investigate the antidepressant effects of PMEOs, depression symptoms were induced by reserpine in mice. The mice were assigned to six groups (*n* = 60): (1) Control group: Not administered with reserpine, PMEOs, or fluoxetine. (2) Negative control (Reserpine) group: Administered with reserpine for 5mg/kg of body weight to induce depression and then did not administer with PMEOs or fluoxetine. (3) Positive control (Fluoxetine) group: Administered with reserpine for 5 mg/kg of body weight to induce depression and then administered with fluoxetine for 20 mg/kg of body weight. (4) Treatment (PMEOs) group: Administered with reserpine for 5 mg/kg of body weight to induce depression and then administered with *P. mariana* extract dissolved in injected sterile normal saline for 50, 100, and 200 mg/kg of body weight [15, 16]. The reserpine was dissolved with 0.8% glacial acetic acid. The corresponding concentrations of PMEOs-L, PMEOs-M, and PMEOs-H groups were 50, 100, and 200 mg/kg, respectively. The drugs were administered intraperitoneally (i.p.). The weight data of the mice were recorded before injection, and the volume of each injection was 0.1 ml per 10 g of mice weight [15]. The groups with reserpine treatment were administered for two weeks, followed by PMEOs or fluoxetine for five days.

### Body Weight and Behavioral Tests

Reserpine Fluoxetine and *P. mariana* at different concentrations were administered 24 h before the test session. Each experimental group consisted of 10 animals.

To measure the effects on the body weight among mice treated with reserpine, fluoxetine, and PMEOs, each group of mice was weighed daily on an electronic scale before administration.

The forced swimming test (FST) was carried out utilizing the Porsolt method (1977) [17]. The mice were placed separately in a rectangular water tank (50 × 30 cm) containing water at a depth of 40 cm for 15 min; the water temperature remained around 23~25°C (pre-test session). Then, the animals were placed in the same experimental conditions after 24 h for 5 min during the test phase. The total immobility time of each mouse was measured during the test session. All of the tests were videotaped and analyzed during the experiment to record the immobility time (in seconds) of each mouse. Mice were considered motionless when they had stopped trying to escape and had only moved enough to maintain their heads above water.

The tail suspension test (TST) was conducted according to the approach Steru *et al*. mentioned (1985) [17]. The distal end of the mouse tail was fixed about 1.5 cm in a cuboid box measuring 25 cm in length and breadth and 60 cm in height for the TST. The head of the mouse was suspended downward without allowing it to touch the surrounding wall. A total of 360 s was recorded, and the measurement index was the immobility time (s) within 240 s after suspension, which reflected the helplessness of mice.

**Fig. 1:**
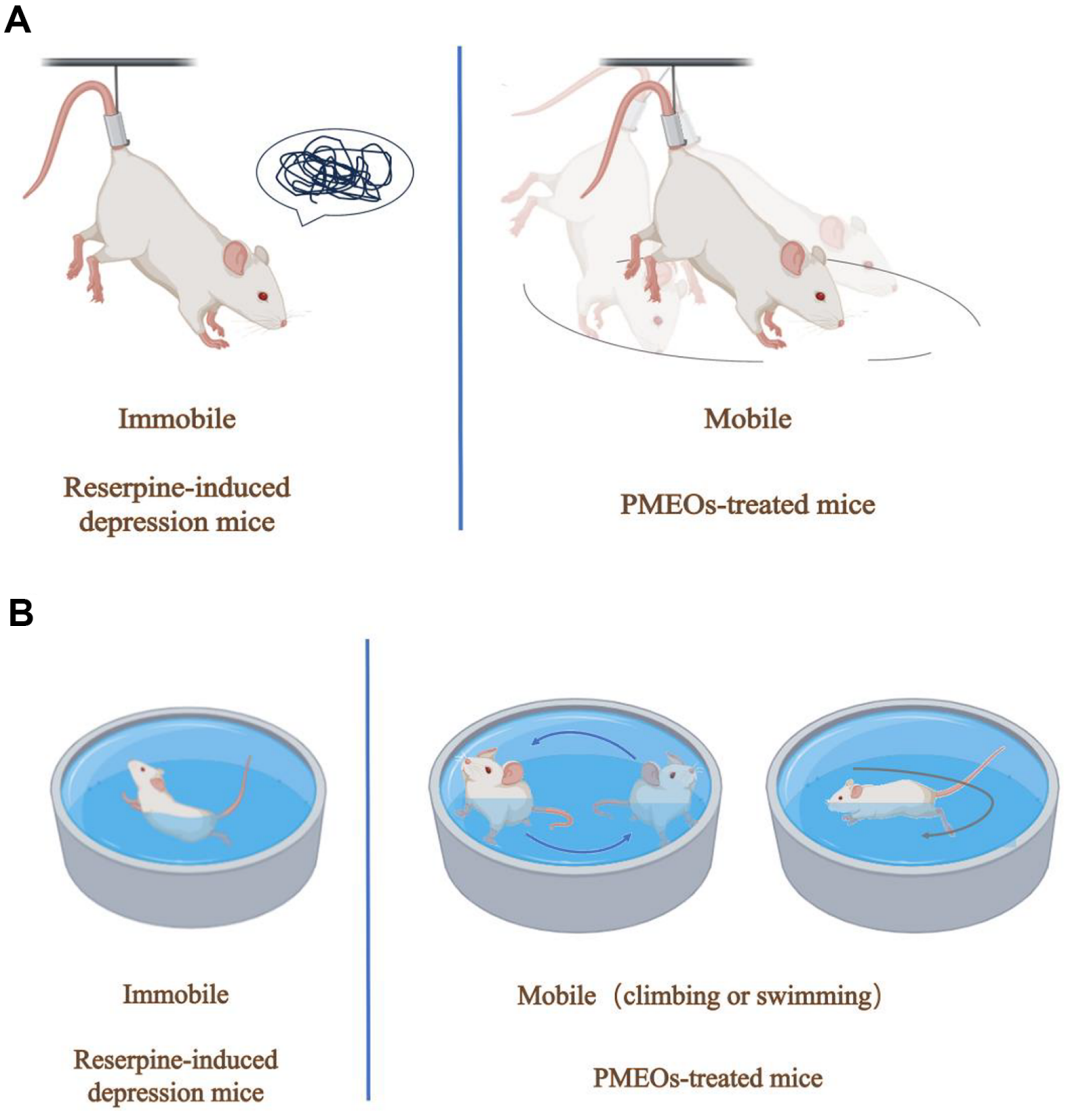
Experimental design representation of tail suspension test (TST) (A) and forced swimming test (FST) (B).

Acclimatize from the mouse living environment to the laboratory environment for at least one hour. The experimental environment was kept quiet to prevent sudden loud noises from panicking the mice to demonstrate that the depression model was effectively induced. The experimental design representation of TST and FST models is shown in Fig. 1.

### Nissl Staining

The brain slices were successively put into xylene I 20 min-xylene II 20 min-anhydrous ethanol I 5 min-anhydrous ethanol II 5 min with 75% alcohol and washed with tap water. After that, all the samples were immersed in paraffin and cut into 5 μm sections with a rotary microtome. They were then dried for one hour at 60°C. Following the deparaffinization of the slices, antigen unmasking was carried out by heating the slices for 10 min in a microwave oven using a sodium citrate buffer (0.01 mol/l; pH 6.0). Following a 10-min immersion in 3% H_2_O_2_ to inhibit endogenous peroxidase activity, tissue slices were blocked for 30 min at room temperature using 10%normal donkey serum. Slices were treated with antibodies for an entire night at 4°C. A fluorescent microscope was used to examine the slides. Images were obtained with the 200× magnification.

### Immunohistochemistry (IHC)

After completing the behavioral tests, mice were sedated with 10% nembutal and immediately sacrificed by decapitation (10 per group). After being dissected, the hippocampal, cortex, and hypothalamus were preserved for 48 h in a 10% neutral formalin fixative. The 5 μm brain slices were fixed in paraffin after being dried in preparation for standard IHC processing. Conventionally, paraffin sections were dewaxed with water and then incubated at room temperature for 10 min with 3% H_2_O_2_. After that, they were rinsed three times for three minutes each with phosphate-buffered solution (PBS). After that, sections were heated to high temperatures utilizing thermal remediation and immersed in citrate buffer salt. Sections were blocked for 20 min at room temperature using 5%normal serum after being cleaned again with PBS. Sections were treated for 15 min at 37°C with horseradish peroxidase enzyme labeled Streptomyces albumen working fluid (1: 100) following an initial 15 min at 37°C incubation with biotinylated anti-rabbit IgG antibody. The DAB (3, 3'-diaminobenzidine) peroxidase substrate kit was used to create the immunostaining reaction, and hematoxylin was used to restain the sample. Sections were dehydrated and then put on glass slides. Software for image analysis, a computer, and a microscope are used for picture processing and acquisition. The hippocampus, cortical, and hypothalamus-positive cells of mice were examined 40 ×10 times under the microscope, respectively. Every mouse was subjected to two discontinuous slices, and from each specimen, three nonoverlapping images were selected at random for statistical analysis and counting.

### Western Blot

After being taken from the animals, the depression-like samples were stored at -80°C and treated for 30 min in RIPA buffer on an ice bath. Ten minutes at 4°C and 12,000 rpm were used to centrifuge the extracted materials. A BCA kit was used to determine the protein level of the supernatant for protein quantification. Each samplés equivalent protein (40 μg) was loaded onto an SDS-PAGE and then moved to a PVDF membrane. After blocking the PVDF membranes with 5% (w/v) BSA in TBST, they were incubated for the entire night at 4°C with anti-5HT-_2A_, anti-CRF, and anti-TrkB (Beyotime Biotechnology Co., China). They were then washed with TBST and probed with certain secondary antibodies conjugated to HRP. A fluorescence scanner detected the band signal, which the electro-chemiluminescence (ECL) kit viewed. Software (FluorChem E, Protein Simple, USA) was used to quantify particular bands, and data was collected, calibrated, and examined.

### Real-Time PCR (RT-qPCR) Analysis

The total RNA of hippocampus samples was extracted using the Trizol reagent (n =3 for each group). Using a Takara PrimeScript First Strand cDNA Synthesis Kit and RNA was reverse transcribed to cDNA. Using SYBR Green (Fermentas), real-time PCR was used to amplify the cDNA. The primer sequences utilized are listed in Table 1, and 60°C was the annealing temperature. The SYBR Green I PCR mix kit was employed to measure the expression of each gene. 20 μl of total volume was used for LightCycler reactions. For every quantitative PCR experiment, three duplicates were carried out. Using the delta−delta Ct technique, the mRNA concentrations of all target genes were adjusted to that of β-actin in each sample. The target result was a two-fold increase in mRNA in the hippocampus compared to control mice. All test kits are used according to the manufacturer's instructions. The primer sequence for RT-qPCR was shown in Table 1.

### Statistical Analysis

One-way analysis of variance (ANOVA) was performed for all data using GraphPad Prism software 8.0.2 (GraphPad Software, USA). Tukey-Kramer multiple comparison test was performed to determine potential significant differences between the control and experimental groups (*p* < 0.05 or *p* < 0.01). R software 4.2.3 (R Foundation for Statistical Computing, Austria) was used for statistical power analysis and to determine sample size with the 'pwr' package. All data are expressed as mean ± SEM and the confidence interval is 95%. The data were obtained in at least three independent experiments.

## Results

### GC-MS Analysis

Herein, the main volatile aromatic compounds and their main components of PMEOs were detected by GC-MS (Table 2). Of the essential oil, 27 effective compounds made up 94.8%, comprising 35.14% monoterpenes, 0.17%oxygenated monoterpenes, and 7.83% sesquiterpenes. The major compounds in PMEOs were (-)-Bornyl acetate (44.95%), γ-Terpinene (14.17%), β-Pinene (10.12%), D-Limonene (9.8%), and α-farnesene (7.87%).

### Effect of PMEOs on Weight Recovery in Depressive-Like Mice

**Fig. 2:**
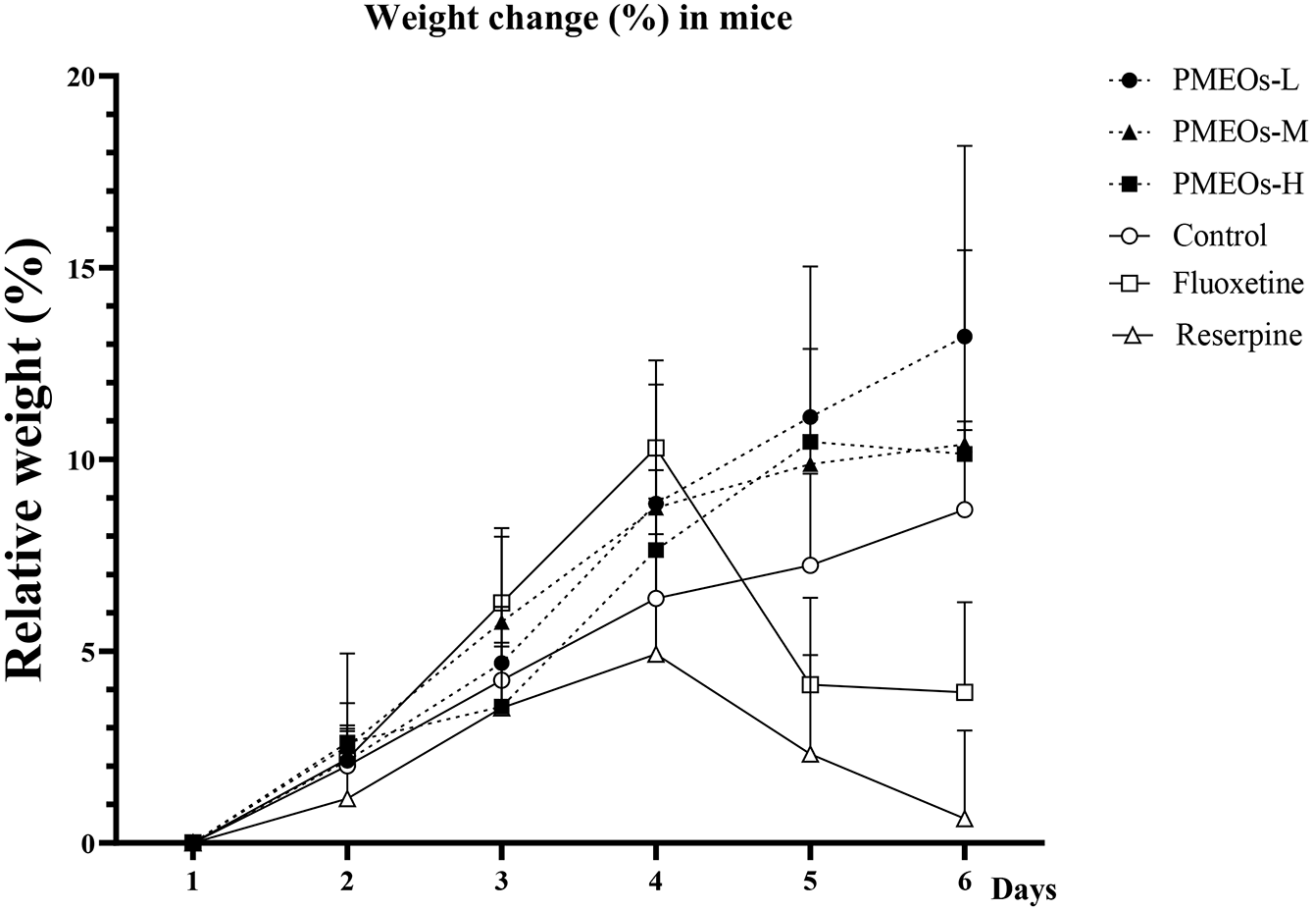
Effect PMEOs on Reserpine-induced mice during 6 days. Data were expressed as the mean ± SEM (*n* = 10).

One of the most common signs of depression is a loss of appetite and weight [18]. Loss of appetite and indigestion caused by the disease can lead to malnutrition and weight loss, which further worsens depression [19]. By summarizing and analyzing the data of weight obtained from the experiment, it was found that the weight of the essential oil group of PMEOs could recover quickly after the weight loss in the modeling group (Fig. 2). Among them, the PMEOs group had a better resistance action to the weight loss caused by reserpine, which means that this essential oil may reverse depression-related dyspepsia and appetite loss. The fluoxetine treatment group lost weight rapidly after 4 days and showed lower weight than the control group. This may be related to the weight fluctuations effect after fluoxetine administration.

### Effect of PMEOs on Reducing the Immobility Time of FST and TST

**Fig. 3:**
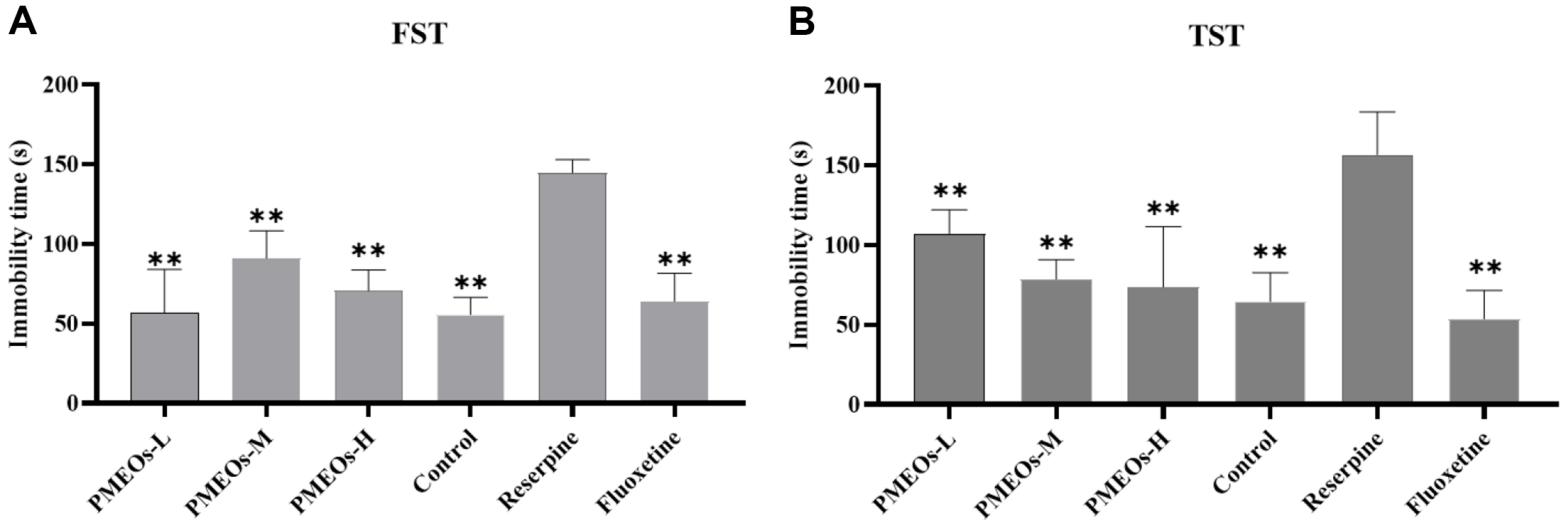
Effect of PMEOs on the immobility time in the FST (A) and the TST (B). The values are shown as mean ± SEM (*n* = 10). The significance of differences from the Reserpine group is at *p < 0.05 and **p<0.01.

The models of FST and TST are frequently used to evaluate the efficacy of antidepressants. These tests were designed to mimic the symptoms of depression in humans by inescapable stressors in mice, and depressive-like mice had a low desire to run away and a long period of inactivity [20, 21]. Under the condition of excluding the interference of external noise or environmental factors, the mice in the reserpine group showed the phenomenon of delayed movement, indicating that the depression model was effectively induced by reserpine. In the FST (Fig. 3A), PMEOs or fluoxetine significantly reduced immobility time relative to the reserpine-treated group (*p* < 0.01) based on the ANOVA. The FST immobility time of PMEOs-L was comparable to that of the control mice. Comparable outcomes might be seen in the TST that PMEOs, especially the PMEOs-H group, significantly decreased the immobility time compared to the reserpine-treated mice (*p* < 0.01) (Fig. 3B). All of these findings pointed to the effectiveness of PMEOs in reducing depressive-like behavior.

### Effects of PMEOs Increased the Normal Neurons

**Fig. 4:**
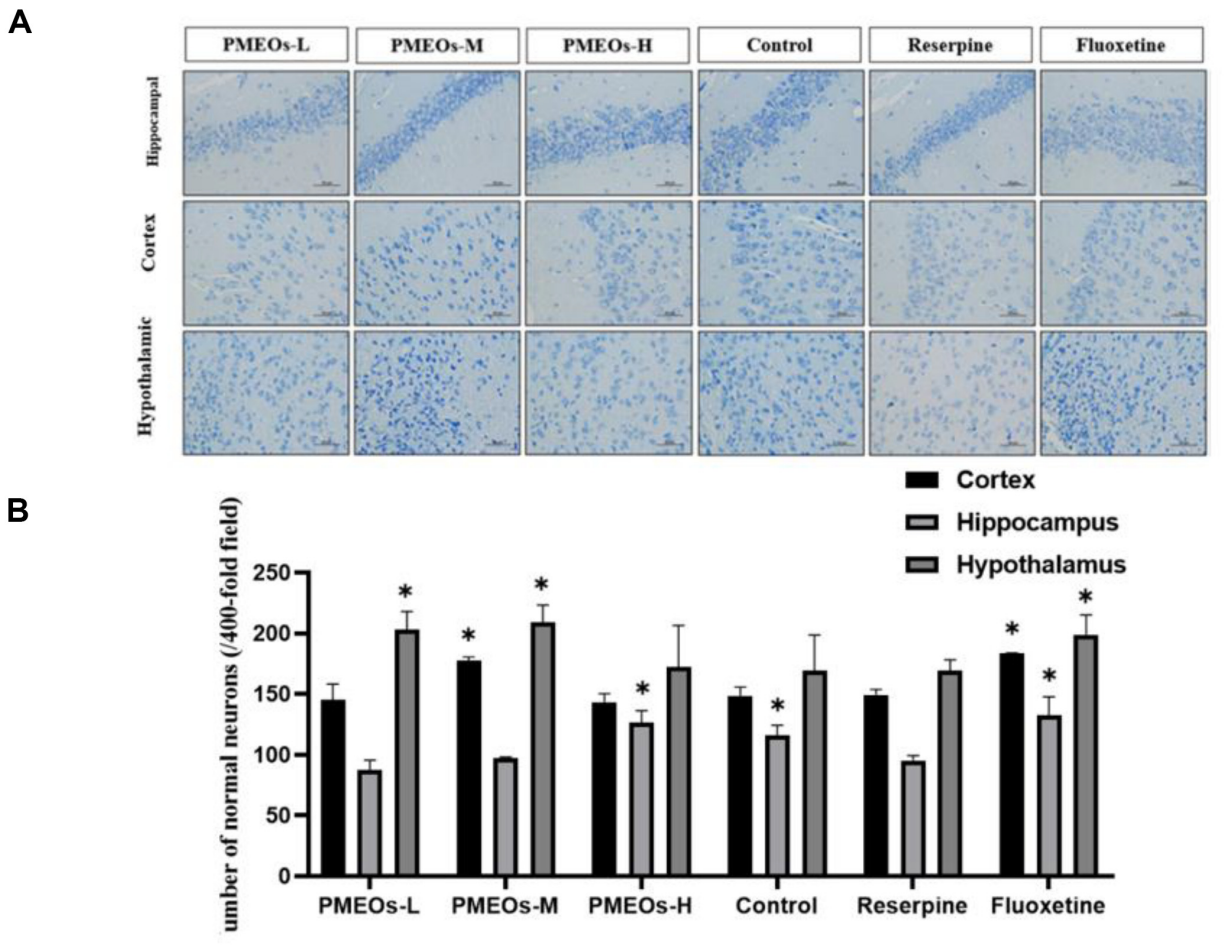
(A) Nissl staining intact cell morphology and effect of PMEOs on Nissl body numbers in hippocampus, cortex and hypothalamus. (B) Nissl-stained neuron cells in the hippocampus, cortex and hypothalamus. Quantification of Nissl bodies in the hippocampus, cortex, and hypothalamus using an image analyzer. IOD integrated optical density. The values are shown as mean ± SEM (*n* = 10). The significance of differences from the Reserpine group is at *p < 0.05 and **p<0.01. (Original magnification × 400, scale bar = 50 μm)

Normal neuronal numbers are essential indicators of depression. Depression can lead to the degeneration of hippocampal neuronal dendrites and a loss of neuronal numbers due to the neurotoxic effects of glucocorticoids [22, 23]. Nissl staining was used to identify surviving neurons and clarify whether PMEOs can reduce neuronal cell death. The Reserpine-treated group presented a marked reduction in the number of neurons, disordered cell arrangements, and shrunken cell bodies in the hypothalamus (Fig. 4A). Compared with the Reserpine group, the PMEOs-treated groups exhibited well-formed, neatly arranged neurons. Neurons in the hippocampal region were higher in the control group than in the reserpine group (*p* < 0.05), but there was no significant difference in neurons in the other two regions. The fluoxetine group showed higher neuron counts in all three regions compared to the reserpine group (*p* < 0.05). After PMEOs-L and PMEOs-M treatments, hypothalamic neuron numbers were notably higher than in the control and reserpine groups (*p* < 0.05). The total of cortical neurons in the PMEOs-M treatment group was significantly higher than that in the reserpine treatment group (*p* < 0.05), while PMEOs-H showed significant therapeutic effects on hippocampus neurons (*p* < 0.05) (Fig. 4B). To sum up, these results suggested that PMEOs could effectively improve neuronal damage.

### IHC Analysis of 5HT-_1A_ and 5HT-_2A_

**Fig. 5:**
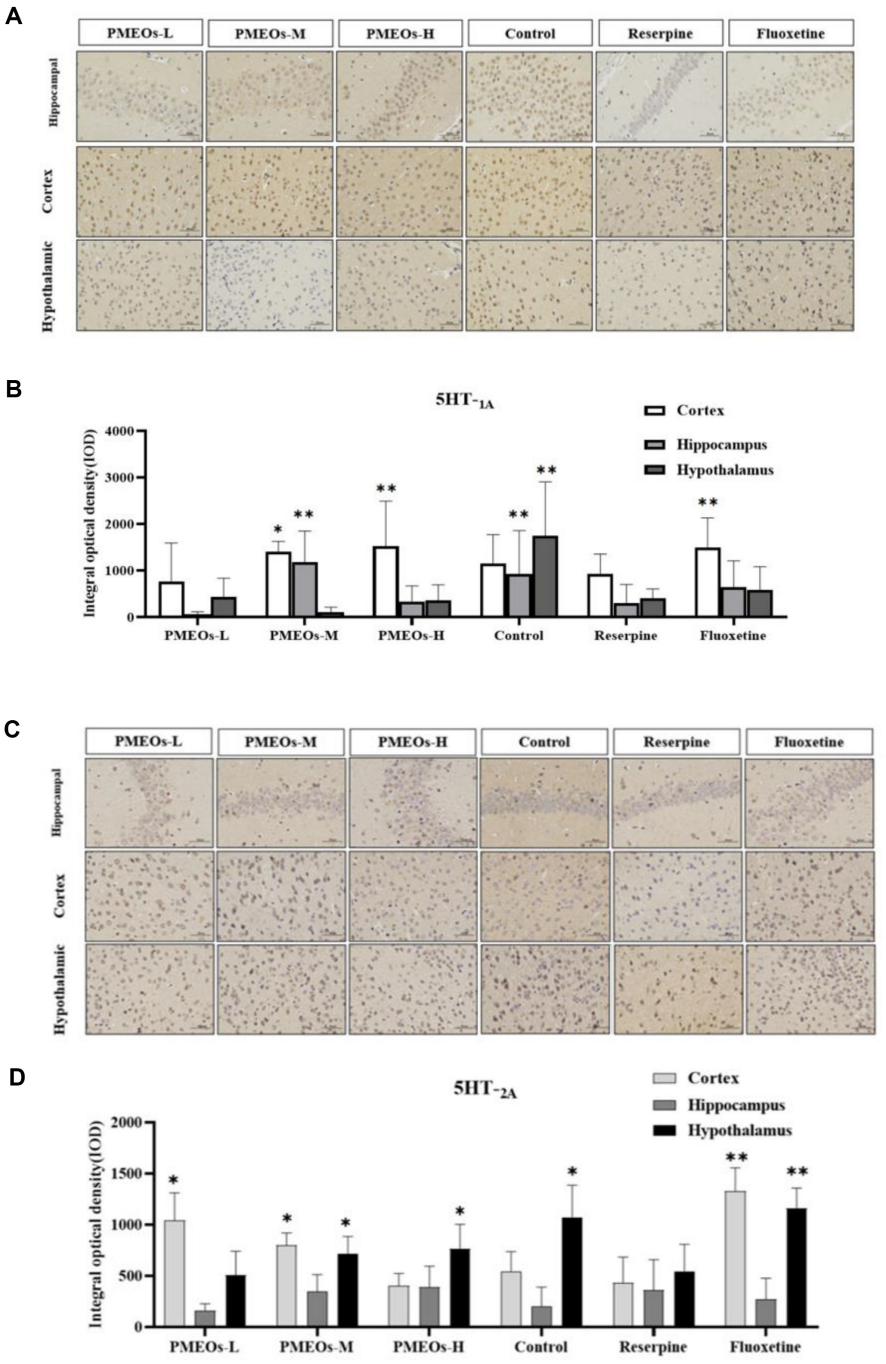
(A-B) Effects of PMEOs on the 5HT-_1A_ expression in the Cortex Hippocampus and Hypothalamus region in Reserpine-induced mice. (C-D) Effects of PMEOs on the 5HT-_2A_ expression in the Cortex Hippocampus and Hypothalamus region in Reserpine-induced mice. The values are shown as mean ± SEM (*n* = 10). The significance of differences from Reserpine group is at *p < 0.05 and **p<0.01.

With lower levels of these receptors in the mouse brain tissue in the reserpine group compared to the control group, the results showed that reserpine-induced depression was linked to disruption in 5HT-_1A_ and 5HT-_2A_ systems. In contrast to the reserpine group, the content of 5HT-_1A_ and 5HT-_2A_ receptors in PMEOs-treated brain tissue was increased, and the cell morphology was excellent and complete (Fig. 5A and 5C).

The number of 5HT-_1A_ receptors was higher in the control and fluoxetine groups compared to the reserpine group, with the control group showing more receptors in the hippocampus and hypothalamus and the fluoxetine group showing more in the cortex. (*p* < 0.01). In terms of the therapeutic effect of PMEOs, the cortical 5HT-_1A_ receptor expression was significantly increased following medium-high dose treatment (*p* < 0.05). Notably, the medium dose of PMEOs restored the number of receptors in the hippocampus to a higher level than in the control group and fluoxetine group, and the same effect was observed in the cortical layer of the high dose of PMEOs (*p* < 0.01) (Fig. 5B).

The number of 5HT-_2A_ in the cortex and hypothalamus was higher in the control and fluoxetine groups compared to the reserpine group, with fluoxetine exhibiting a more pronounced effect. (*p* < 0.01). Interestingly, a similar effect was observed in the PMEOs treatment group. Low doses restored 5-HT2A receptors in the cortex, while high doses targeted the hypothalamus. (*p* < 0.05). Moderate doses of PMEOs exerted notable impacts on both the cortical and hypothalamic regions. (*p* < 0.05) (Fig. 5D).

In summary, PMEOs increase the content of 5HT-_1A_ and 5HT-_2A_ in brain tissue, which can regulate the balance of nerve receptors in the brain, reduce the overactivation state of the central nervous system, and alleviate depressive symptoms.

### Western Blotting and Relative Densities of 5HT-_2A_, CRF and TrkB

**Fig. 6:**
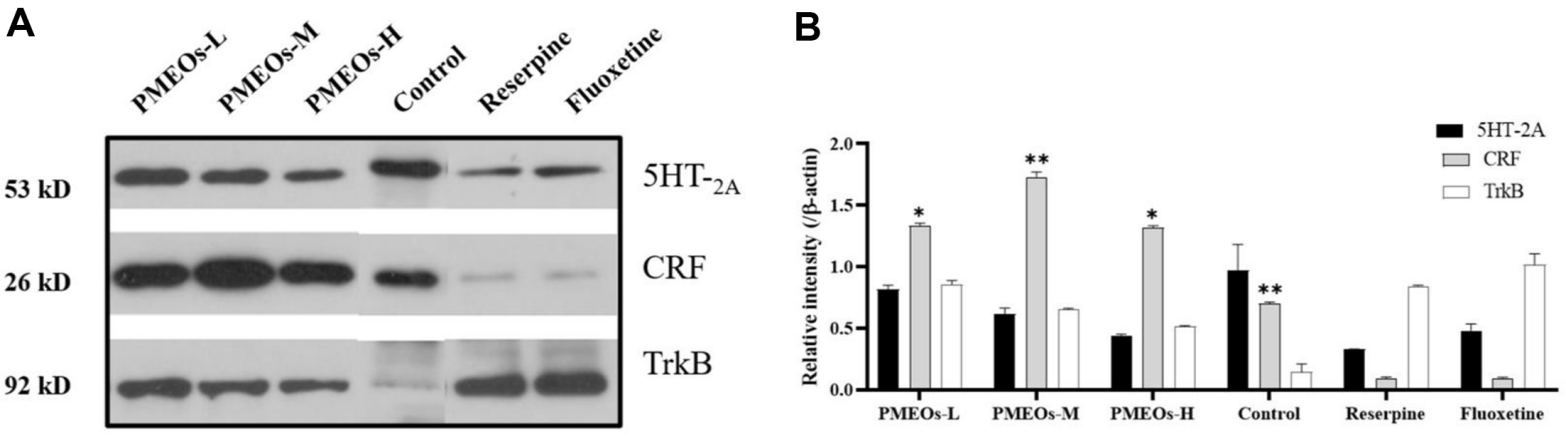
Effect of administration of PMEOs on the expression of 5HT-_2A_, CRF and TrkB in the brain of Reserpine-induced mice. (**A**) Western blotting and (**B**) Relative densities of 5HT-_2A_, CRF and TrkB. The values are shown as mean ± SEM (*n* = 10). The significance of differences from Reserpine group is at *p < 0.05 and **p<0.01.

To assess the antidepressant potential of PMEOs in a reserpine-induced depression model, western blotting was performed to measure the levels of 5HT-_2A_, CRF, and TrkB protein expression in whole brain tissue homogenates. Results As shown in Fig. 6A & 6B below, the expression of 5HT-_2A_ and CRF in the reserpine treatment group decreased, while the expression of TrkB increased. Compared with the reserpine treatment group, both the PMEOs and fluoxetine treatment group upregulated the expression of 5HT-_2A_. The level of CRF was increased, and the level of TrkB was down-regulated in all groups of PMEOs (*p* < 0.05). Moreover, the TrkB levels decreased as the dose of PMEOs was reduced, while 5HT-_2A_ expression increased. This suggests a better antidepressant effect with lower doses of PMEOs. The results imply that the antidepressant and immunomodulatory effect of PMEOs may be achieved by affecting the 5HT-_2A_, CRF and TrkB protein pathways.

### RT-qPCR Detected the Relative mRNA Level of 5HT-_1A_, 5HT-_2A_, BDNF, CRF and TrkB

**Fig. 7:**
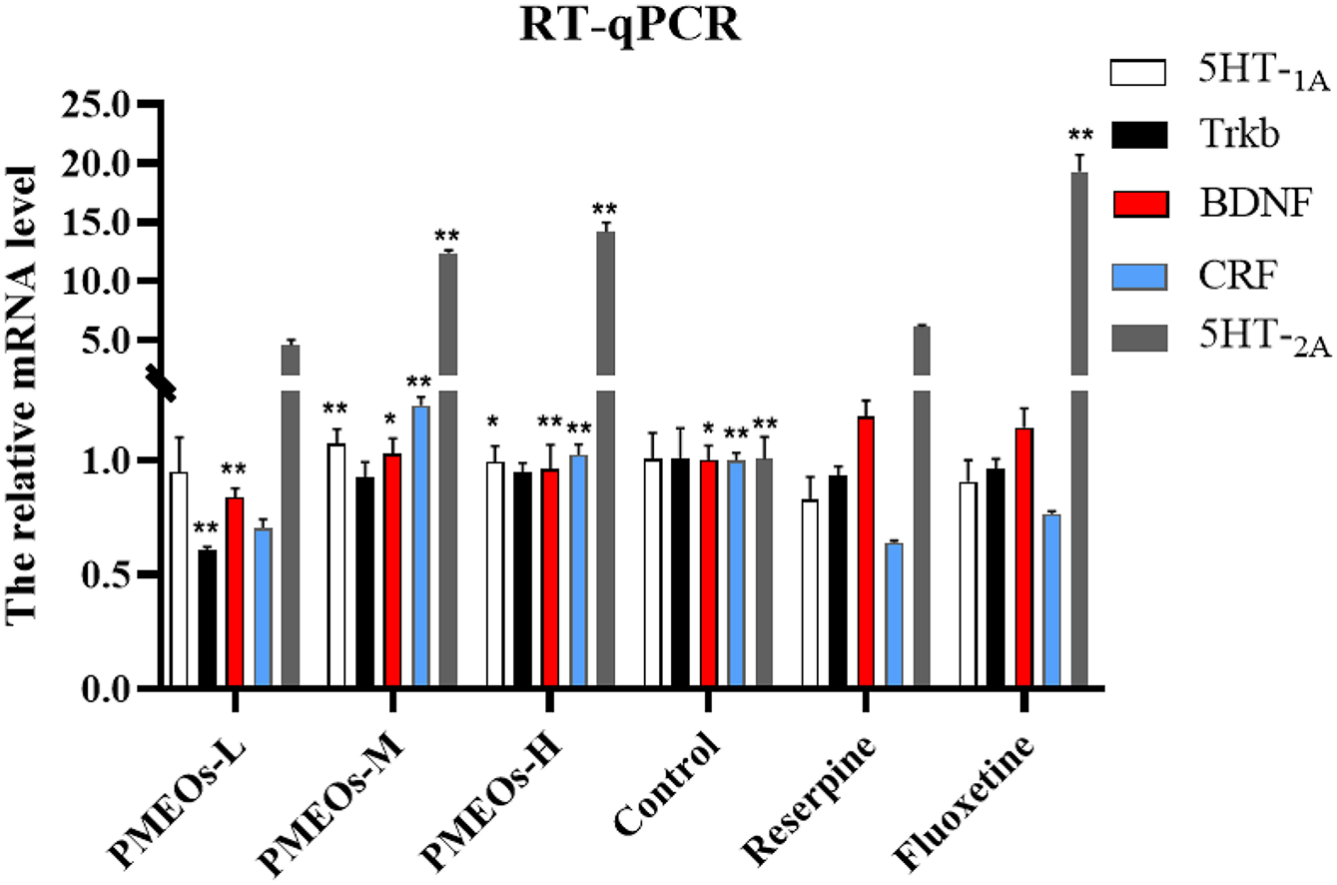
The relative mRNA level of 5HT-_1A_, TrkB, BDNF, CRF, and 5HT-_2A_ by RT-qPCR. The values are shown as mean ± SEM (*n* = 10). The significance of differences from Reserpine group is at *p < 0.05 and **p < 0.01.

To comprehensively evaluate the regulatory mechanism of PMEOs on depression-related neurotransmitters, RT-qPCR was performed to examine the mRNA levels of 5HT-_1A_, 5HT-_2A_, TrkB, BDNF, and CRF in whole brain tissue homogenates. Compared with the reserpine group, mRNA relative expression levels of CRF and 5HT-_1A_ in PMEOs-H and PMEOs-M brain tissues were increased (*p* < 0.01), while BDNF decreased (*p* < 0.05) (Fig. 7). The relative mRNA expression levels of BDNF and TrkB in PMEOs-L were significantly decreased (*p* < 0.01). The relative mRNA expression of 5HT-_2A_ in PMEOs-M, PMEOs-H and fluoxetine groups showed a significant upward trend compared with that of reserpine (*p* < 0.01).

## Discussion

Current research believes that the nervous system regulates the immune system through neurotransmitters, and depression is a common disease of nervous system immune disorder [24, 25]. Compared to synthetic or semi-synthetic drugs, natural extracts treat depression with fewer side effects and are widely popular. The effective components of *P. mariana* have been isolated and identified [26, 27]. However, the research on the relationship between *P. mariana* and the central nervous system is still insufficient, especially the mechanism for treating depressive behavior needs to be further explored. Reserpine-induced acute depression is one of the commonly used methods to construct depression models. It can cause a series of depressive physiological changes in bradykinesia and hypothermia through consuming monoamine neurotransmitters, such as serotonin (5-HT), dopamine (DA) and noradrenaline (NA) [28]. Fluoxetine is one of the most commonly used medications for depression. In this study, reserpine was used to induce depression in mice, and fluoxetine was used as a positive drug. Different doses of PMEOs (50,100 and 200mg/kg) were set up to compare their antidepressant effects. The primary aim of this study was to examine the mechanism of PMEOs' effects on depressive behavior in mice, along with investigating the correlation between PMEOs and the expression of relevant neurotransmitters and proteins in brain tissue.

The water extract of *P. mariana* was found to be rich in polyphenol compounds. Resveratrol and proanthocyanidins have been reported in water-extracted *P. mariana* [26, 29]. However, the main components of PMEOs were identified by GC-MS, which included (-) -bornyl acetate (44.95%), γ-Terpinene (14.17%), and β-Pinene (10.12%). As the main component, β-pinene may have a significant improvement in depressive symptoms. The β-pinene and γ-Terpinene are both monoterpene compounds, which can affect mood by directly acting on the central nervous system and olfactory nerves [30]. Notably, pinene has a low molecular weight and high lipophilicity, which can affect neurotransmitters across the blood-brain barrier. The β-Pinene is used to treat depression and anxiety by targeting 5-HT1A and beta-adrenergic receptor subtypes [31]. The bornyl acetate was reported to have been found in valerian extract as a stimulant for 5-HT receptors [32]. Together, these primary components promote the balance of neurotransmitters in the brain and maintain a normal emotional state.

Weight loss and slow movement are momentous symptoms of depression. Herein, weight change test, TST, and FST tests were used to evaluate depressive behavior and the effectiveness of reserpine modeling in mice. The mice treated with reserpine showed significant negative behavior, suggesting the effectiveness of the reserpine-induced depression model. After fluoxetine treatment, mice weights remained below that of the control group, whereas PMEOs treatment led to weight gains surpassing the control group. Low-dose PMEOs outperformed fluoxetine in FST, and the therapeutic effect of PMEOs in TST intensified with higher concentrations. These indicated that PMEOs can effectively improve the low struggle desire and anorexia caused by depression in mice.

Nissl body is a kind of basophilic substance in the cytoplasm of neurons, which exists widely in various neurons. When neurons are affected by injury or disease, the number, distribution, and morphology of the nissl body change. High doses of PMEOs exerted therapeutic effects on hippocampus neurons, medium doses on the cortex and hypothalamus, and low doses primarily on the hypothalamus. PMEOs can reverse the decline in the number of normal neurons in the brain of mice, reducing neuronal atrophy or death.

The hypothalamic-pituitary-adrenal axis (HPA) is an integral part of the neuroendocrine system, involved in controlling the response to stress and regulating many physical activities, such as the immune system, mood, and energy storage and expenditure. Serotonin (5-HT) is a monoamine neurotransmitter in the brain. When serotonin levels in the brain dip below average, it can lead to depression. IHC tests showed that moderate and high doses of PMEOs activated cortical 5HT-_1A_ receptors on par with fluoxetine, and medium doses even surpassed fluoxetine in activating the receptors in the hippocampus. Fluoxetine upregulated 5HT-_2A_ receptors significantly in the cortex and hypothalamus, and PMEOs also mainly enhanced the receptor expression in these regions. The protective mechanism of PMEOs on the nervous system may be related to the regulation of the levels of 5HT-_1A_ and 5HT-_2A_ in different sites by different doses. It is speculated that PMEOs can activate the 5-HT receptor in the brain, thus producing an inhibitory effect on the secretion of ACTH. Maintaining HPA axis balance prevents depressive symptoms stemming from its hyperfunction.

In the HPA axis, the hypothalamic paraventricular nucleus (PVN) releases CRF and stimulates the anterior pituitary to release adrenocorticotropic hormone (ACTH). In turn, this stimulates the adrenal gland to release glucocorticoids (*e.g.*, cortisol), generating negative feedback that impacts the cortex, hippocampus, and hypothalamus. Elevated cortisol levels lead to BDNF expression being suppressed, which results in neuronal death and depression [33]. Upon binding to BDNF, TrkB, its crucial receptor, triggers signaling pathways that boost neuronal synaptic plasticity, supporting enhanced neuronal function. Elevating BDNF expression or activating TrkB receptors can promote neuronal growth and survival, ultimately easing symptoms for depression patients. In this study, western blotting was used to evaluate the effects of PMEOs on critical proteins such as 5HT, CRF, and TrkB. Western blotting results showed that the decreased expression of 5HT-_2A_ and CRF in the reserpine treatment group was associated with depletion of monoamine neurotransmitters or imbalance of their secretion. The expression of 5HT-_2A_ can be upregulated in the PMEOs treatment group, and PMEOs may play an antidepressant role by increasing the 5-HT level or enhancing signal conduction from 5-HT. In addition, CRF expression was elevated in the PMEOs treatment group, which may be related to the regulatory effect of PMEOs on the HPA axis to enhance the adaptability of the body to stress response. The RT-qPCR revealed an overall increase in CRF, 5HT-_1A_, and 5HT-_2A_ expression after PMEOs treatment, notably a significant rise in 5HT-_2A_ receptor levels with medium and high doses.

In the current study, it has been confirmed that increases in BDNF and TrkB have a positive effect on alleviating neurotransmitter imbalances caused by excess acetylcholine [14]. However, it is important to note that an abnormally active state of acetylcholinergic neurons in the brain may be also related to the pathogenesis of depression [34]. In recent years, anti-muscarinic drugs such as atropine and scopolamine have also been included in the research scope of depression treatment strategies [35-37]. In this paper, PMEOs treatment further reduced the expression of BDNF and its receptor TrkB in reserpin-induced depressed animals compared to the control group. In the physiological state, the expression of various enzymes and receptors in the body is maintained in a dynamic equilibrium state. In view of this, excess acetylcholine may upset this equilibrium state, and PMEOs may restore this equilibrium by reducing the expression of BDNF and TrkB.

In summary, as an effective natural antidepressant, *P. mariana* combats depression by regulating the balance of central neurotransmitters and proteins in the brain that have specific neurological functions. However, this study only discussed the therapeutic effects of different doses of PMEOs on depressive-like mice, and the degree of dose-dependent effects and the interaction between various neurotransmitters still need to be further studied.

## Figures and Tables

**Table 1:** Primer sequence for RT-qPCR.

Gene	Sequence of primer (5’→3’)	
	Sense	Antisense
5HT-_1A_	CCAACTATCTCATCGGCTCCTT	CTGACCCAGAGTCCACTTGTTG
5HT-_2A_	TATGCTGCTGGGTTTCCTTGT	GTTGAAGCGGCTATGGTGAAT
TrkB	AACGGAGACTACACCCTGATGG	GCAATCACCACCACGGCATA
BDNF	TATTAGCGAGTGGGTCACAGCG	TATTAGCGAGTGGGTCACAGCG
CRF	AGCCCTTGAATTTCTTGCAGC	GCAGCGGGACTTCTGTTGAG

**Table 2:** Retention index (RI) and relative content (%) of each chemical isolated from PMEOs.

No	Compounds ^i^	RT ^ii^	RI ^iii^	Exp.RI	Ref. ^iV^	Relative content (%)
1	β-Pinene	3.143	796	980	A	10.12
2	γ-Terpinene	3.603	819	1059	A	14.17
3	D-Limonene	3.886	833	1028	G	9.8
4	allo-Ocimene	6.208	925	1132	E	0.12
5	Terpinolen	5.089	893	1081	H	0.93
6	β-Elemen	17.621	1193	1389	J	0.18
7	Longifolene	17.984	1203	1414	A	0.45
8	Caryophyllene	18.451	1219	1423	C	0.31
9	β-Selinene	20.452	1285	1487	B	0.1
10	Selinene	20.72	1294	1495	B	0.12
11	Muurolene	20.928	1301	1501	A	0.86
12	γ-Cadinene	21.316	1316	1511	I	1.51
13	δ-Cadinene	21.63	1328	1522	B	4.09
14	α-Cadinene	21.919	1339	1538	F	0.21
15	L-Pinocarveol	6.661	936	1144	G	0.17
16	Linalool	5.313	902	1095	H	0.45
17	Fenchol	5.801	915	1097	A	0.1
18	camphenehydrate	7.028	946	1148	F	0.22
19	Borneol	7.749	964	1150	H	1.4
20	Terpinen-4-ol	8.228	976	1183	A	0.46
21	α-terpineol	8.971	995	1199	A	1.55
22	Fenchylacetate	10.469	1026	1217	J	0.41
23	(-)-Bornyl acetate	14.168	1099	1289	D	44.95
24	Geranylacetate	17.444	1188	1363	H	0.22
25	T-Muurolol	24.577	1444	1640	I	0.98
26	τ-Cadinol	24.661	1448	1639	I	0.14
27	α-Cadinol	24.9	1458	1653	F	0.78
Total identified/%						94.8
Total monoterpenes/%						35.14
Total oxygen monoterpenes/%						0.17
Total sesquiterpenes/%						7.83
Others/%						51.66

^i^The compounds eluted from methyl silicone capillary columns (30 m × 0.25 mm, 0.25-μm film thickness) are arranged sequentially.

^ii^Retention time (RT).

^iii^Retention index (RIs) of normal alkanes (C_6_-C_40_) on the same methyl silicone capillary column.

^iV^Literature index. ^A^Nellie Francezon and Tatjana Stevanovic, 2017; ^B^Bouddah Poaty *et al*., 2015; ^C^Nellie Francezon, 2018; ^D^Sen-Sung Cheng *et al*., 2008; ^E^ Alessandra Bertol *et al*., 2011; ^F^Robert P. Adams *et al*., 2005; ^G^M. A. Ferhat *et al*., 2007; ^H^Gianna Allegron *et al*., 2006; ^I^Mohamed Hazzit *et al*., 2006; ^J^T. Dob, D. Dahmane and C. Chelghoum, 2008.

## References

[1] Giannelli FR (2020). Major depressive disorder. JAAPA.

[2] Tao X, Chi O, Delaney PJ, Li L, Huang J (2021). Detecting depression using an ensemble classifier based on quality of life scales. Brain Inform..

[3] Dhama K, Sharun K, Gugjoo MB, Tiwari R, Alagawany M, Iqbal Yatoo M (2023). A comprehensive review on chemical profile and pharmacological activities of *Ocimum basilicum*. Food Rev. Int..

[4] Murrough JW, Abdallah CG, Mathew SJ (2017). Targeting glutamate signalling in depression: progress and prospects. Nat. Rev. Drug Discov..

[5] Nisca A, Ștefănescu R, Stegăruș DI, Mare AD, Farczadi L, Tanase C (2021). Phytochemical profile and biological effects of spruce (Picea abies) bark subjected to ultrasound assisted and microwave-assisted extractions. Plants (Basel).

[6] Boivin M, Bourdeau N, Barnabé S, Desgagné-Penix I (2021). Black spruce extracts reveal antimicrobial and sprout suppressive potentials to prevent potato (*Solanum tuberosum* L.) losses during storage. J. Agric. Food Chem..

[7] Tang M, Ai Y, Song N, Geng L, Ren L, Zhu S (2023). Chemical composition and hypnotic effect of *Picea mariana* essential oils. J. Essent. Oil Bear. Pl..

[8] accouch R, Rascol E, Stoklosa K, Alves ID (2022). The role of the lipid environment in the activity of G protein coupled receptors. Biophys. Chem..

[9] Tafet GE, Nemeroff CB (2020). Pharmacological treatment of anxiety disorders: the role of the HPA axis. Front. Psychiatry.

[10] De Gregorio D, Enns JP, Nuñez NA, Posa L, Gobbi G (2018). d-Lysergic acid diethylamide, psilocybin, and other classic hallucinogens: mechanism of action and potential therapeutic applications in mood disorders. Prog. Brain Res..

[11] Malcolm B, Thomas K (2022). Serotonin toxicity of serotonergic psychedelics. Psychopharmacology (Berl).

[12] Jiang Y, Peng T, Gaur U, Silva M, Little P, Chen Z (2019). Role of corticotropin releasing factor in the neuroimmune mechanisms of depression: examination of current pharmaceutical and herbal therapies. Front. Cell. Neurosci..

[13] Kim JE, Chae S, Kim S, Jung YJ, Kang MG, Heo W (2021). Cerebellar 5HT-2A receptor mediates stress-induced onset of dystonia. Sci. Adv..

[14] Machaalani R, Chen H (2018). Brain derived neurotrophic factor (BDNF), its tyrosine kinase receptor B (TrkB) and nicotine. Neurotoxicology.

[15] Shahamat Z, Abbasi-Maleki S, Mohammadi Motamed S (2016). Evaluation of antidepressant-like effects of aqueous and ethanolic extracts of Pimpinella anisum fruit in mice. Avicenna J. Phytomed..

[16] Ghaderi H, Rafieian M, Nezhad HR (2018). Effect of hydroalcoholic *Cinnamomum zeylanicum* extract on reserpine-induced depression symptoms in mice. Pharmacophore.

[17] Poleszak E, Szopa A, Bogatko K, Wyska E, Wośko S, Świąder K (2019). Antidepressant-like activity of typical antidepressant drugs in the forced swim test and tail suspension test in mice is augmented by DMPX, an adenosine A 2A receptor antagonist. Neurotox. Res..

[18] Simmons WK, Burrows K, Avery JA, Kerr KL, Taylor A, Bodurka J (2020). Appetite changes reveal depression subgroups with distinct endocrine, metabolic, and immune states. Mol. Psychiatry.

[19] Akilimali PZ, Musumari PM, LundimuTugirimana P, Beya P, Mutombo FJ, Kayembe PK (2016). Depressive symptoms, loss of appetite and under nutrition among treated HIV patients: a cross sectional study in Goma, the Democratic Republic of Congo. J. Nutr. Health Food Sci..

[20] Kraeuter AK, Guest PC, Sarnyai Z (2019). The forced swim test for depression-like behavior in rodents. Methods Mol. Biol..

[21] Fitzgerald PJ, Yen JY, Watson BO (2019). Stress-sensitive antidepressant-like effects of ketamine in the mouse forced swim test. PLoS One.

[22] Ruiz NAL, Del Ángel DS, Olguín HJ, Silva ML (2018). Neuroprogression: the hidden mechanism of depression. Neuropsychiatr. Dis. Treat..

[23] Lim DW, Park J, Jung J, Kim SH, Um MY, Yoon M, Kim YT (2020). Dicaffeoylquinic acids alleviate memory loss via reduction of oxidative stress in stress-hormone-induced depressive mice. Pharmacol. Res..

[24] Won E, Kim YK (2016). Stress, the autonomic nervous system, and the immune-kynurenine pathway in the etiology of depression. Curr. Neuropharmacol..

[25] Fung TC, Olson CA, Hsiao EY (2017). Interactions between the microbiota, immune and nervous systems in health and disease. Nat. Neurosci..

[26] Francezon N, Stevanovic T (2017). Chemical composition of essential oil and hydrosol from *Picea mariana* bark residue. BioResources.

[27] Álvarez-Chávez BJ, Godbout S, Le Roux É, Palacios JH, Raghavan V (2019). Bio-oil yield and quality enhancement through fast pyrolysis and fractional condensation concepts. Biofuel Res. J..

[28] Strawbridge R, Javed RR, Cave J, Jauhar S, Young AH (2023). The effects of reserpine on depression: a systematic review. J. Psychopharmacol..

[29] Diouf PN, Stevanovic T, Cloutier A (2009). Study on chemical composition, antioxidant and anti-inflammatory activities of hot water extract from Picea mariana bark and its proanthocyanidin-rich fractions. Food Chem..

[30] Zhang LL, Yang ZY, Fan G, Ren JN, Yin KJ, Pan SY (2019). Antidepressant-like effect of *Citrus sinensis* (L.) Osbeck essential oil and its main component limonene on mice. J. Agric. Food Chem..

[31] Weston-Green K, Clunas H, Jimenez Naranjo C (2021). A review of the potential use of pinene and linalool as terpene-based medicines for brain health: discovering novel therapeutics in the flavours and fragrances of cannabis. Front. Psychiatry.

[32] Shinjyo N, Waddell G, Green J (2020). Valerian root in treating sleep problems and associated disorders-A systematic review and meta-analysis. J. Evid. Based.Integr. Med..

[33] Masuo Y, Satou T, Takemoto H, Koike K (2021). Smell and stress response in the brain: review of the connection between chemistry and neuropharmacology. Molecules (Basel, Switzerland).

[34] Mitić M, Lazarević-Pašti T (2021). Does the application of acetylcholinesterase inhibitors in the treatment of Alzheimer's disease lead to depression?. Expert Opin. Drug Metab. Toxicol..

[35] Siqueira AA, Cunha AF, Marques GLM, Felippe ISA, Minassa VS, da Silva Gramelich TC (2019). Atropine counteracts the depressive-like behaviour elicited by acute exposure to commercial chlorpyrifos in rats. Neurotoxicol. Teratol..

[36] Kolar D (2021). Scopolamine as a potential treatment option in major depressive disorder-A literature review. Isr. J. Psychiatry..

[37] Drevets WC, Bhattacharya A, Furey ML (2020). The antidepressant efficacy of the muscarinic antagonist scopolamine: past findings and future directions. Adv. Pharmacol..

